# Alteration in TET1 as potential biomarker for immune checkpoint blockade in multiple cancers

**DOI:** 10.1186/s40425-019-0737-3

**Published:** 2019-10-17

**Authors:** Hao-Xiang Wu, Yan-Xing Chen, Zi-Xian Wang, Qi Zhao, Ming-Ming He, Ying-Nan Wang, Feng Wang, Rui-Hua Xu

**Affiliations:** 1 Department of Medical Oncology, State Key Laboratory of Oncology in South China, Collaborative Innovation Center for Cancer Medicine, Sun Yat-sen University Cancer Center, Guangzhou, 510060 China; 2 Department of Experimental Research, State Key Laboratory of Oncology in South China, Collaborative Innovation Center for Cancer Medicine, Sun Yat-sen University Cancer Center, Guangzhou, 510060 China

**Keywords:** Biomarker, DNA methylation, Immune checkpoint blockade, Pan-cancer, *TET1*

## Abstract

**Background:**

Immune checkpoint inhibitors (ICIs) have achieved impressive success in different cancer types, yet responses vary and predictive biomarkers are urgently needed. Growing evidence points to a link between DNA methylation and anti-tumor immunity, while clinical data on the association of genomic alterations in DNA methylation-related genes and ICI response are lacking.

**Methods:**

Clinical cohorts with annotated response and survival data and matched mutational data from published studies were collected and consolidated. The predictive function of specific mutated genes was first tested in the discovery cohort and later validated in the validation cohort. The association between specific mutated genes and tumor immunogenicity and anti-tumor immunity was further investigated in the Cancer Genome Altas (TCGA) dataset.

**Results:**

Among twenty-one key genes involving in the regulation of DNA methylation, *TET1*-mutant (*TET1*-MUT) was enriched in patients responding to ICI treatment in the discovery cohort (*P* < 0.001). *TET1* was recurrently mutated across multiple cancers and more frequently seen in skin, lung, gastrointestinal, and urogenital cancers. In the discovery cohort (*n* = 519), significant differences were observed between *TET1*-MUT and *TET1*-wildtype (*TET1*-WT) patients regarding objective response rate (ORR, 60.9% versus 22.8%, *P* < 0.001), durable clinical benefit (DCB, 71.4% versus 31.6%, *P* < 0.001), and progression-free survival (PFS, hazard ratio = 0.46 [95% confidence interval, 0.25 to 0.82], *P* = 0.008). In the validation cohort (*n* = 1395), significant overall survival (OS) benefit was detected in the *TET1*-MUT patients compared to *TET1*-WT patients (hazard ratio = 0.47 [95% confidence interval, 0.25 to 0.88], *P* = 0.019), which was, importantly, independent of tumor mutational burden and high microsatellite instability; as well as not attributed to the prognostic impact of *TET1*-MUT (*P* > 0.05 in both two non-ICI-treated cohorts). In TCGA dataset, *TET1*-MUT was strongly associated with higher tumor mutational burden and neoantigen load, and inflamed pattern of tumor-infiltrating T lymphocytes, immune signatures and immune-related gene expressions.

**Conclusions:**

*TET1*-MUT was strongly associated with higher ORR, better DCB, longer PFS, and improved OS in patients receiving ICI treatment, suggesting that *TET1*-MUT is a novel predictive biomarker for immune checkpoint blockade across multiple cancer types.

## Background

Immune checkpoint inhibitors (ICIs) that target cytotoxic T lymphocyte antigen 4 (CTLA-4) or the programmed cell death (ligand) 1 [PD-(L)1] pathway have achieved impressive success in the treatment of different cancer types [1]. However, clinical responses vary, and biomarkers predictive of response may help to identify patients who will derive the greatest therapeutic benefit [2].

PD-L1 expression, high microsatellite instability (MSI-H), tumor mutational burden (TMB), T cell-inflamed gene expression profile (GEP), and specific mutated genes were reported to exhibit predictive utility in identifying responders to ICI treatment [1, 3–7]. However, only PD-L1 and MSI-H have been clinically validated hitherto [2]. Therefore, more predictive biomarkers are in urgent need.

Growing evidence points to a link between DNA methylation and anti-tumor immunity/immunotherapy [8–10]. For instance, changes in DNA methylation level have been found to correlate with the degree of immune infiltration of the tumor [11]; and DNA methylation signature was recently reported to be associated with the efficacy of anti-PD-1 treatment in non-small-cell lung cancer (NSCLC) [12]. However, to date, clinical evidence on the association of genomic alterations in DNA methylation-related genes and ICI response are lacking.

In this study, we systematically collected and consolidated a large amount of genomic and clinical data to evaluate the predictive function of mutations in key genes involving in the regulation of DNA methylation [13, 14]. And we found that mutations in *TET1*, a DNA demethylase, was predictive of higher objective response rate (ORR), better durable clinical benefit (DCB), longer progression-free survival (PFS) and improved overall survival (OS) to ICI treatment.

## Methods

### Pan-cancer alteration frequency analysis

For determination of the alteration frequency of *TET1* among cancer types, all the genomic data from the curated set of non-redundant studies on the cBioPortal (https://www.cbioportal.org) [15, 16] were collected and processed as shown in Additional file 1: Figure S1. Tumors with nonsynonymous somatic mutations in the coding region of *TET1* was defined as *TET1*-mutant (*TET1*-MUT), while tumors without as *TET1*-wildtype (*TET1*-WT) [7].

### Clinical cohort consolidation

**Fig. 1:**
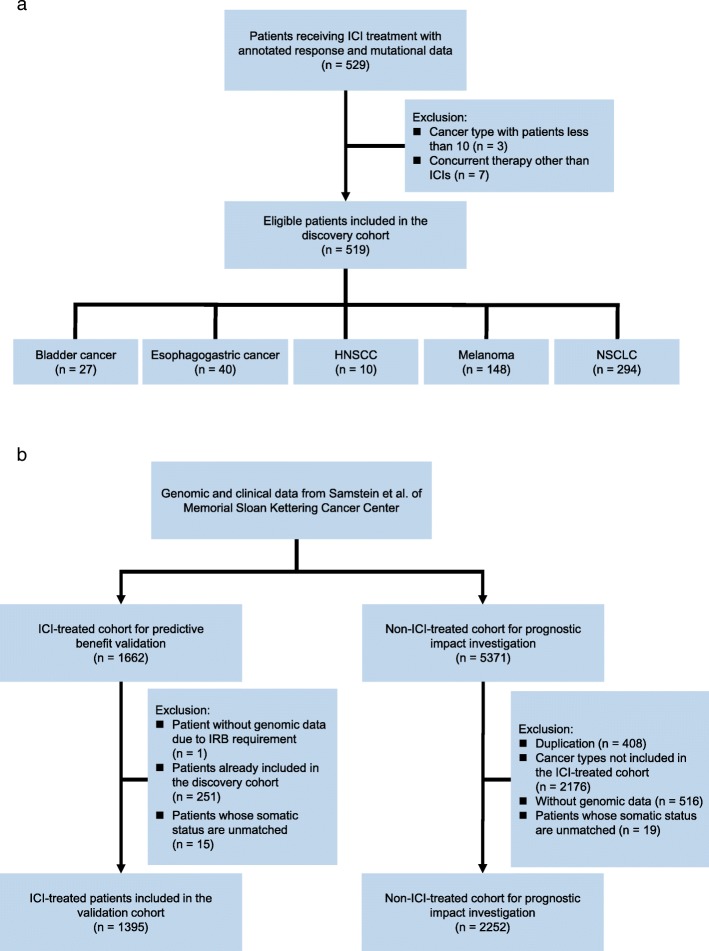
Flowchart of the clinical cohort consolidation. **a**. Consolidation of the discovery cohort from six published studies. **b**. Consolidation of the validation cohort and the non-ICI-treated cohort from Samstein et al. ICI, immune checkpoint inhibitor; HNSCC, head and neck squamous cell carcinoma; NSCLC, non-small-cell lung cancer; IRB, Institutional Review Board

To evaluate the predictive function of specific mutated genes to ICI treatment, a discovery cohort with annotated response and mutational data of patients receiving ICI treatment from six published studies [17–22] was collected and consolidated (Fig. 1a). Samples of the first two cohorts [17, 18] (*n* = 280) were sequenced using MSK-IMPACT panel, a U.S. Food and Drug Administration (FDA) authorized comprehensive genomic profiling panel. While whole-exome sequencing (WES) was applied to samples of the latter four cohorts [19–22] (*n* = 249), which were previously curated and filtered by excluding records without response data and records without qualified mutational data by Miao et al. [22]. Based on Miao et al.’s efforts, we further excluded records of cancer type with patients less than 10 (*n* = 3) and patients receiving concurrent therapy besides ICIs (*n* = 7). In the end, 519 patients from five cancer types were included in the discovery cohort. An expanded ICI-treated cohort from Samstein et al. [23] without response data but with survival data was used as the validation cohort to further validated the predictive function of *TET1*-MUT to ICI treatment (Fig. 1b). The non-ICI-treated cohort from Samstein et al. [23] was also included to investigate the possibility that the observed survival differences among patients with *TET1*-MUT and *TET1*-WT might simply be attributable to a general prognostic benefit of *TET1*-MUT, unrelated to ICIs. As the non-ICI-treated cohort from Samstein et al. mainly consisted of patients with advanced disease, the Cancer Genome Altas (TCGA) cohort consisting of 20 cancer types with adequate survival information as determined by Liu et al. [24] was additionally employed to investigate the prognostic impact of *TET1*-MUT.

### TMB normalization in the clinical cohorts

TMB was defined as the total number of nonsynonymous somatic, coding, base substitution, and indel mutations per megabase (Mb) of genome examined [25]. For samples sequenced by WES, the total number of nonsynonymous mutations were normalized by megabases covered at adequate depth to detect variants with 80% power using MuTect given estimated tumor purity, as determined by Miao et al. [22]. For samples sequenced by MSK-IMPACT panel, the total number of nonsynonymous mutations identified was normalized to the exonic coverage of the MSK-IMPACT panel (0.98, 1.06, and 1.22 Mb in the 341-, 410-, and 468-gene panels, respectively), and mutations in driver oncogenes were not excluded from the analysis as Samstein et al. proposed [23]. As previously described, the cutoff of the top 20% within each histology was used to divided patients into TMB-high and TMB-low groups [23].

### Clinical outcomes

The primary clinical outcomes were ORR, DCB, PFS, and OS. ORR was assessed using Response Evaluation Criteria in Solid Tumors (RECIST) version 1.1. DCB was classified as durable clinical benefit (DCB; complete response [CR]/partial response [PR] or stable disease [SD] that lasted > 6 months) or no durable benefit (NDB, progression of disease [PD] or SD that lasted ≤6 months) [18]. Patients who had not progressed and were censored before 6 months of follow-up were considered not evaluable (NE). PFS was assessed from the date the patient began immunotherapy to the date of progression or death of any cause. Patients who had not progressed were censored at the date of their last scan. In the discovery cohort and validation cohort, OS was calculated from the start date of ICI treatment, and patients who did not die were censored at the date of last contact. Notably, in the non-ICI-treated cohort from Samstein et al., OS was calculated from the date of first infusional chemotherapy, while in the TCGA cohort, OS was calculated from the date of first diagnosis.

### Tumor immunogenicity and anti-tumor immunity analysis

To characterize the tumor immune microenvironment of *TET1*-MUT tumors, we further compared the TMB, neoantigen load, tumor-infiltrating leukocytes, immune signatures and immune-related gene expressions between *TET1*-MUT and *TET1*-WT tumors in the TCGA dataset. Somatic mutational data from Ellrott et al. [26], neoantigen data from Thorsson et al. [27], and RNA-seq data from UCSC Xena data portal (https://xenabrowser.net) were collected and processed as shown in Additional file 2: Figure S2. TMB was retained as the total number of somatic nonsynonymous mutation count without normalization, and neoantigen load was defined as the total predicted neoantigen count as determined by Thorsson et al. [27]. The R package MCP-counter was used to estimate the abundance of tumor-infiltrating leukocytes [28]. Seven classical immune signatures were adopted from Rooney et al. [29], and the R package GSVA was used to determine the single sample gene set enrichment (ssGSEA) scores of each immune signature [30]. Immune-related genes and their functional classification were obtained from Thorsson et al. [27], whose expression level was quantified as FPKM (Fragments Per Kilobase of exon model per Million mapped fragments) and log2-transformed.

### Statistical analysis

Assessment of enrichment of specific mutated genes with response (CR/PR versus PD/SD) was done with fisher’s exact test and the *P* values were controlled for false discovery rate (FDR). The association between *TET1* status and ORR or DCB were examined using fisher’s exact test. The progression-free and overall survival probability of *TET1*-MUT and *TET1*-WT patients were analyzed by Kaplan–Meier method, log-rank test, and Cox proportional hazards regression analysis, which were adjusted for available confounding factors, including 1) age, sex, cancer type, and TMB in the discovery cohort; 2) age, sex, cancer type, TMB status, and MSI status in the validation cohort; 3) sex, cancer type, TMB status in the non-ICI-treated cohort from Samstein et al.; and 4) age, sex, ethnicity, cancer type, histology grade, tumor stage, TMB, and year of first diagnosis in the TCGA cohort. Interactions between the *TET1* status and the following factors were assessed, including age (≤ 60 versus > 60 years), sex (male versus female), cancer type (melanoma, bladder cancer, NSCLC versus other cancers), TMB status (low versus high) and drug class (monotherapy versus combination therapy). The differences of TMB, neoantigen load, tumor-infiltrating leukocytes, immune signatures, or immune-related gene expressions between *TET1*-MUT and *TET1*-WT tumors were examined using Mann-Whitney U test. The nominal level of significance was set at 0.05 and all statistical tests were two-sided. Statistical analyses were performed using R v. 3.5.2 (http://www.r-project.org).

## Results

### *TET1*-MUT was enriched in patients responding to ICI treatment

**Fig. 2:**
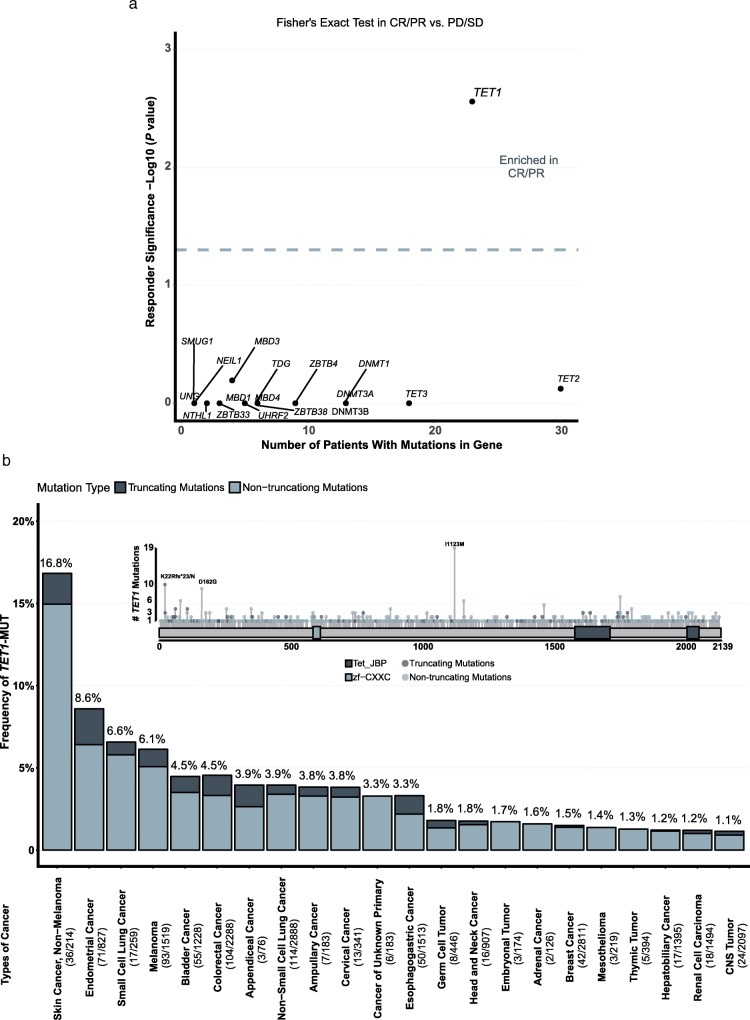
*TET1*-MUT was enriched in patients responding to ICI treatment. **a**. Response-associated mutations in CR/PR versus PD/SD (two-tailed Fisher’s exact test, *n* = 126 patients with CR/PR, *n* = 389 patients with PD/SD). The dashed grey line indicated false discovery rate adjusted *P* = 0.05 (Fisher’s exact test). **b**. The proportion of *TET1*-MUT tumors identified for each cancer type with alteration frequency above 1%. Numbers above the barplot indicated the alteration frequency, numbers close to cancer names indicated the number of *TET1*-MUT patients and the total number of patients. ‘CNS tumor’ denoted tumor in the central nervous system. ‘Truncating mutations’ included nonsense, nonstop, splice site mutations, and frameshift insertion and deletion; ‘non-truncating mutations’ included missense mutations and inframe insertion and deletion

As shown in Fig. 1a, mutational data with annotated response data were pooled from six publicly available studies to form the discovery cohort, which included 519 patients across five cancer types: bladder cancer (*n* = 27), esophagogastric cancer (*n* = 40), head and neck squamous cell carcinoma (HNSCC) (*n* = 10), melanoma (*n* = 148), and NSCLC (*n* = 294). Patient characteristics of the discovery cohort were summarized in Table 1. Particularly, more than half (61.7%) of patients were treated with PD-(L)1 monotherapy, representing its predominant role in immunotherapy. Twenty-one key genes involving in the regulation of DNA methylation, including DNA methyltransferase *DNMT1*, *DNMT3A*, *DMNT3B*, and DNA demethylase *TET1*, *TET2*, *TET3*, and other mediators, were manually collected from previous studies [13, 14] (Additional file 3: Table S1) and investigated. Within these genes, *TET1*-MUT was significantly enriched in patients responding to ICI treatment (Fig 2a) (*P* = 0.003), indicating that *TET1*-MUT may be a potential predictive biomarker for ICI treatment.

**Table 1:** Patient characteristics of the discovery cohort

Characteristics	No. (%)
No. of patients	519
Median age, years (range)	64 (18–92)
Sex	
Male	300 (57.8)
Female	219 (42.2)
Cancer type	
Bladder cancer	27 (5.2)
Esophagogastric cancer	40 (7.7)
Head and neck cancer	10 (1.9)
Melanoma	148 (28.5)
Non-small-cell lung cancer	294 (56.6)
Drug class	
CTLA-4, monotherapy	142 (27.4)
PD-(L)1, monotherapy	320 (61.7)
CTLA-4 + PD-(L)1, combination therapy	57 (11.0)
Best overall response	
CR/PR	126 (24.3)
SD	137 (26.4)
PD	252 (48.6)
NE	4 (0.8)
Durable clinical benefit	
DCB	165 (31.8)
NDB	330 (63.6)
NE	24 (4.6)
Median TMB, Mutation/Mb (IQR)	7.14 (3.77–13.24)
*TET1* status	
Mutant	23 (4.4)
Wildtype	496 (95.6)

Abbreviations: *CR* complete response, *CTLA-4* cytotoxic T-cell lymphocyte-4, *DCB* durable clinical benefit, *IQR* interquartile range, *Mb* megabase, *NDB* no durable benefit, *NE* not evaluable, *PD* progressive disease, *PD-(L)1* programmed cell death-1 or programmed death-ligand 1, *PR* partial response, *SD* stable disease, *TMB* tumor mutational burden

There were 23 *TET1*-MUT patients, accounting for 4.4% of the population in the discovery cohort (Table 1). We further investigated the alteration frequency of *TET1* across multiple cancer types with genomic data collected from cBioportal. After data assembling, 32,568 patients from 39 cancer types were included in the analysis (Additional file 1: Figure S1). The somatic mutations of *TET1* were evenly distributed (Fig. 2b), without any annotated functional hotspot mutations from 3D Hotspots [31] (https://www.3dhotspots.org). The average alteration frequency of *TET1* was 2.4% among these 39 cancer types, 22 of which had an alteration frequency above 1%. Skin, lung, gastrointestinal tract and urogenital system were among the most frequently affected organs (Fig. 2b).

### Association of *TET1* status and clinical outcomes in the discovery cohort

**Fig. 3:**
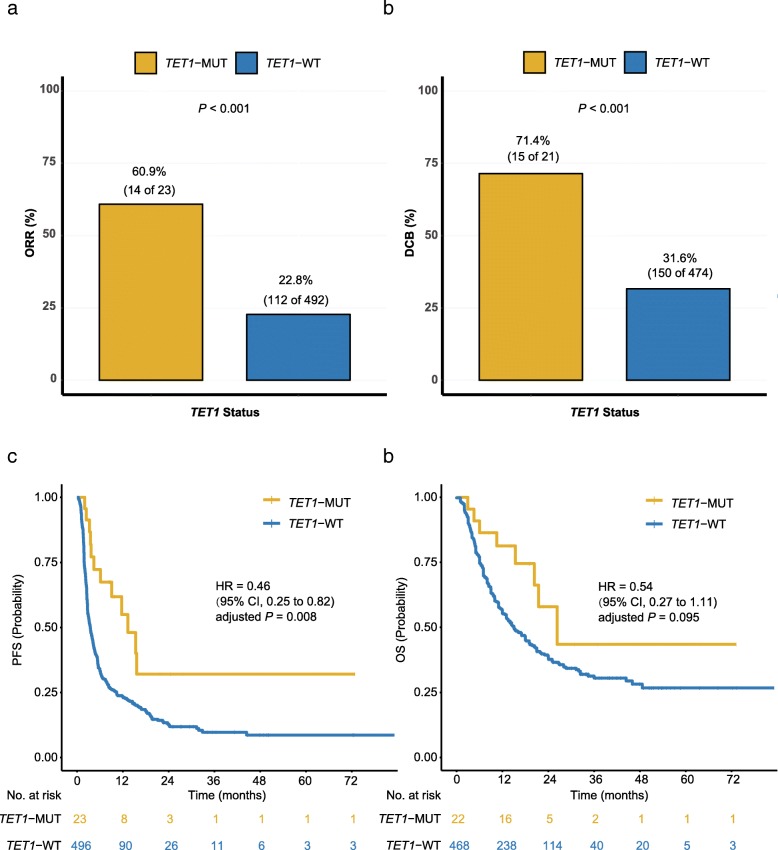
Association of *TET1* status and clinical outcomes in the discovery cohort. **a**. Histogram depicting proportions of patients achieved objective response (ORR) in *TET1*-MUT and *TET1*-WT groups. (*n* = 515; 4 patients with best overall response not evaluable). **b**. Histogram depicting proportions of patients derived durable clinical benefit (DCB) in *TET1*-MUT and *TET1*-WT groups. (*n* = 495; 24 patients with durable clinical benefit not evaluable). **c**. Kaplan-Meier estimates of progression-free survival (PFS) in the discovery cohort comparing patients with *TET1*-MUT with their respective WT counterparts. (*n* = 519). **d**. Kaplan-Meier estimates of overall survival (OS) in the discovery cohort comparing patients with *TET1*-MUT with their respective WT counterparts. (*n* = 490; 29 patients with no OS information available)

The baseline patient characteristics according to *TET1* status were shown in Additional file 4: Table S2, and no significant differences were observed between *TET1*-MUT and *TET1*-WT patients except for TMB. According to RECIST version 1.1, the best overall response of four patients was not evaluable. In the remaining 515 patients, 23 patients were *TET1*-MUT while 492 patients were *TET1*-WT. The ORR of patients with *TET1*-MUT was more than 2.5-fold higher than that of patients with *TET1*-WT (Fig. 3a, 60.9% versus 22.8%, odds ratio = 5.26 [95% confidence interval (CI), 2.06 to 14.16], *P* < 0.001). As for DCB, 71.4% (15/21) of patients with *TET1*-MUT derived DCB from ICI treatment; while only 31.6% (150/474) of patients with *TET1*-WT did (Fig. 3b, odds ratio = 5.38 [95% CI, 1.93 to 17.27], *P* < 0.001). As expected, longer PFS, adjusted by age, sex, cancer type and TMB, of patients with *TET1*-MUT was observed (Fig. 3c, hazard ratio [HR] = 0.46 [95% CI, 0.25 to 0.82], adjusted *P* = 0.008). The median PFS was 13.32 months (95% CI, 9.10 to not reached) in the *TET1*-MUT group versus 3.49 months (95% CI, 2.99 to 4.05) in the *TET1*-WT group. The OS benefit from ICI treatment was also more prominent in the *TET1*-MUT group than that in the *TET1*-WT group (Fig. 3d, median OS, 26.4 months [95% CI, 20.3 to not reached] in the *TET1*-MUT group versus 15.0 months [95% CI, 13.0 to 18.2] in the *TET1*-WT group). However, after adjusted for age, sex, cancer type, and TMB, there was only numerically significant OS benefit (HR = 0.54 [95% CI, 0.27 to 1.11], adjusted *P* = 0.095), probably due to the limited sample size of the *TET1*-MUT group (*n* = 22).

### Validation of the predictive function of *TET1*-MUT

**Fig. 4:**
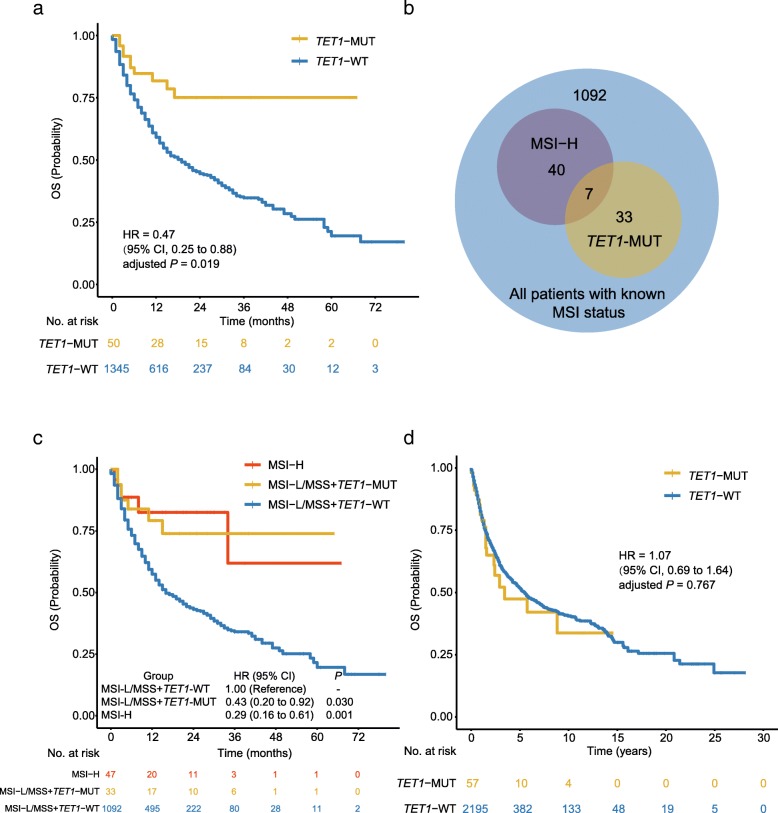
Validation of the predictive function of *TET1*-MUT. **a**. Kaplan-Meier curves comparing the overall survival (OS) of patients with *TET1*-MUT and patients with *TET1*-WT in the validation cohort. **b**. Venn diagram showing the concomitant presence of MSI-H and TET1-MUT within patients with known MSI status of the validation cohort. **c**. Kaplan-Meier curves comparing the OS in the MSI-H, MSI-L/MSS and *TET1*-MUT, and MSI-L/MSS and *TET1*-WT groups in the validation cohort. **d**. Kaplan-Meier curves investigating the prognostic impact of *TET1*-MUT in the non-ICI-treated cohort from Samstein et al.

**Fig. 5:**
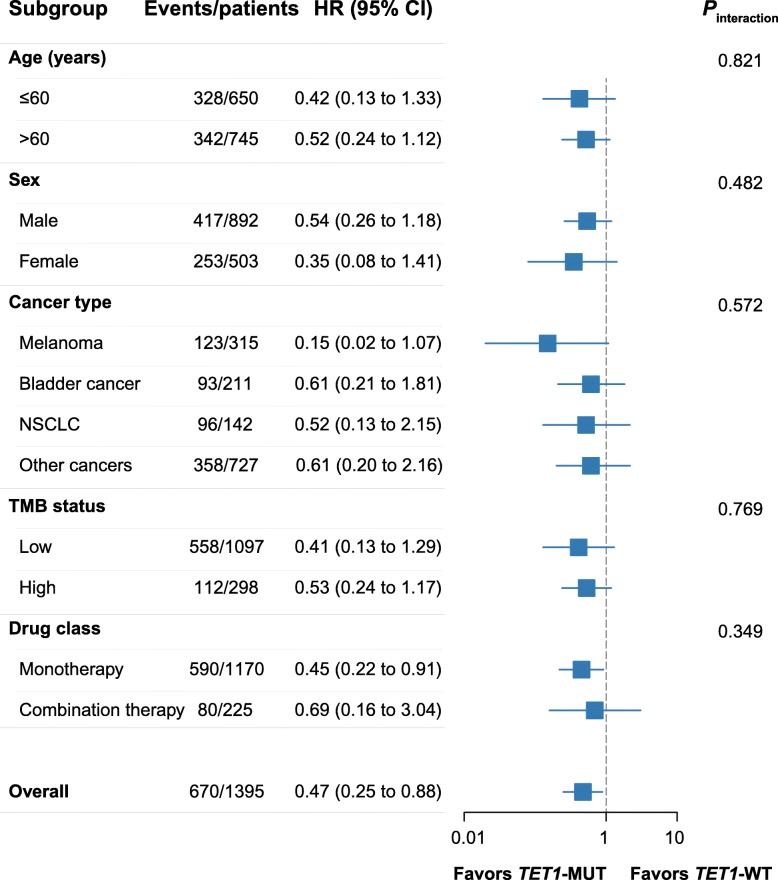
Stratification analysis in subgroups of age, sex, cancer type, TMB status and drug class in the validation cohort. Breast cancer, colorectal cancer, esophagogastric cancer, glioma, head and neck cancer, renal cell carcinoma and cancer of unknown primary were combined into ‘other cancers’ as the *TET1*-MUT cases or deaths were insufficient for hazard ratio calculation in each single cancer type. ‘Monotherapy’ indicated monotherapy of CTLA-4, PD-1 or PD-L1 antibody; ‘combination therapy’ indicated combination therapy of CTLA-4 with PD-(L)1 antibodies

To further validate the predictive function of *TET1*-MUT on OS benefit, an expanded ICI-treated cohort (*n* = 1395) was gathered (Fig. 1b). In this validation cohort, the OS benefit was still more prominent in the *TET1*-MUT group than that in the *TET1*-WT group (Fig. 4a, the median OS was not reached in the *TET1*-MUT group versus 19.0 months [95% CI, 16.0 to 22.0] in the *TET1*-WT group). Even after adjusted for confounding factors, including age, sex, cancer type, MSI status and TMB status, *TET1*-MUT still independently predicted favorable OS outcomes (Fig. 4a, HR = 0.47 [95% CI, 0.25 to 0.88], adjusted *P* = 0.019). In patients with known MSI status (*n* = 1172), 47 of them were MSI-H while 40 were *TET1*-MUT, and only 7 patients were both MSI-H and *TET1*-MUT (Fig. 4b). Notably, in patients with low microsatellite instability (MSI-L) or microsatellite stable (MSS), *TET1*-MUT could still identify patients whose OS was significantly longer than that of *TET1*-WT patients (Fig. 4c, HR = 0.43 [95% CI, 0.20 to 0.92], adjusted *P* = 0.030), and nearly equal to that of MSI-H patients (Fig. 4c), indicating that *TET1*-MUT was compatible and comparable with MSI-H as predictive biomarkers. The favorable clinical outcomes for *TET1*-MUT versus *TET1*-WT were also prominent and consistent across subgroups of age, sex, cancer type, TMB status and drug class (Fig. 5, all *P*_interaction_ > 0.05).

To confirm that the OS benefit from ICI treatment in patients with *TET1*-MUT was not simply attributed to its general prognostic impact, we further evaluated the survival differences between *TET1*-MUT and *TET1*-WT patients in two non-ICI-treated cohorts. Survival difference was observed between patients with *TET1*-MUT and patients with *TET1*-WT neither in the non-ICI-treated cohort from Samstein et al. (Fig. 4d, n = 2252, HR = 1.07 [95% CI, 0.69 to 1.64], adjusted *P* = 0.767), nor in the TCGA cohort (Additional file 5: Figure S3, *n* = 6035, HR = 1.14 [95% CI, 0.91 to 1.42], adjusted *P* = 0.261).

### Association of *TET1*-MUT with enhanced immunogenicity and activated anti-tumor immunity

**Fig. 6:**
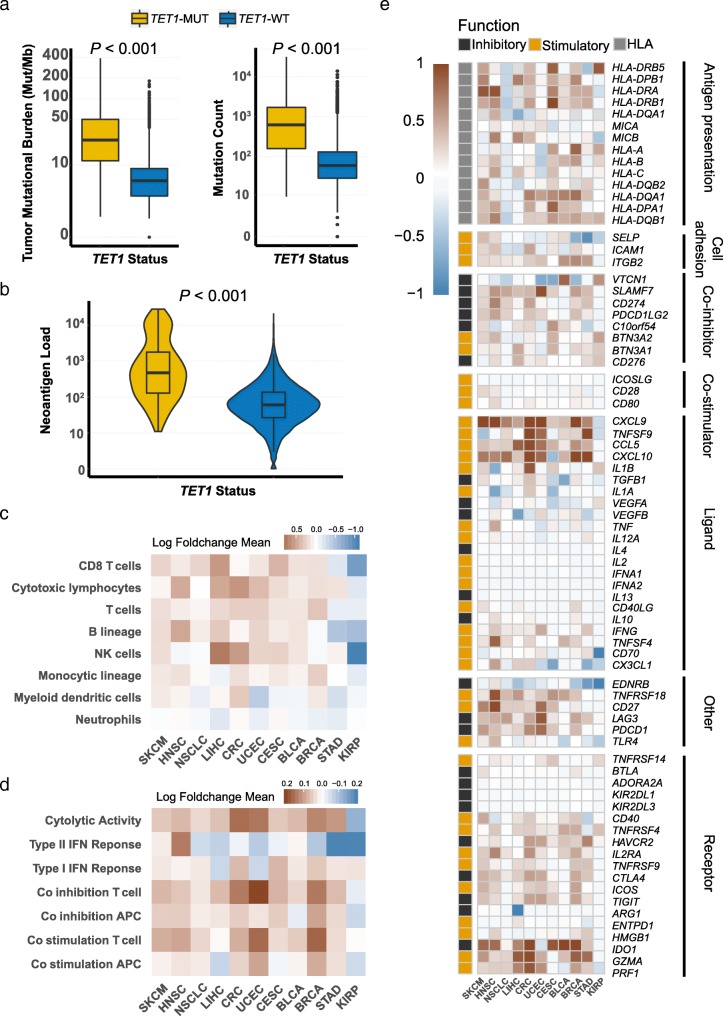
*TET1*-MUT was associated with enhanced tumor immunogenicity and activated anti-tumor immunity. **a**. Comparison of tumor mutational burden between the *TET1*-MUT and *TET1*-WT tumors in the Samstein’s cohort (left panel) and the TCGA cohort (right panel). **b**. Comparison of neoantigen load between the *TET1*-MUT and *TET1*-WT tumors in the TCGA cohort. **c**. Heatmap depicting the log2-transformed fold change in the mean tumor-infiltrating leukocytes MCP-counter scores of the *TET1*-MUT tumors compared to *TET1*-WT tumors across different cancer types. **d**. Heatmap depicting the log2-transformed fold change in the mean immune signature ssGSEA scores of the *TET1*-MUT tumors compared to *TET1*-WT tumors across different cancer types. **e**. Heatmap depicting the mean differences in immune-related gene mRNA expressions between *TET1*-MUT and *TET1*-WT tumors across different cancer types. The x-axis of the heatmap indicated different cancer types and the y-axis indicated tumor-infiltrating leukocytes, immune signatures, or gene names. Each square represented the fold change or difference of each indicated tumor-infiltrating leukocyte, immune signature, or immune-related gene between *TET1*-MUT and *TET1*-WT tumors in each cancer type. Red indicated up-regulation while blue indicated down-regulation

To characterize the tumor immune microenvironment of *TET1*-MUT tumors, we compared the tumor immunogenicity and anti-tumor immunity between *TET1*-MUT and *TET1*-WT tumors. The TMB level was significantly higher in *TET1*-MUT tumors compared with that in the *TET1*-WT tumors, both in the Samstein’s cohort (Fig. 6a, left panel, *P* < 0.001) and in the TCGA cohort (Fig. 6a, right panel, *P* < 0.001). Accordantly, the neoantigen load was also significantly higher in *TET1*-MUT tumors (Fig. 6b, *P* < 0.001), indicating that *TET1*-MUT was associated with enhanced tumor immunogenicity.

On the other hand, the tumor-infiltrating T lymphocytes, especially cytotoxic lymphocytes, were generally more abundant in the *TET1*-MUT tumors compared with those in the *TET1*-WT tumors across multiple cancer types (Fig. 6c, Additional file 6: Figure S4). Besides, the results of the immune signature analysis showed that cytolytic activity signal was also significantly higher in the *TET1*-MUT tumors, along with general upregulation of modulatory signals, including co-inhibitory and co-stimulatory factors on antigen-presenting cells and T cell (Fig. 6d). To better characterize the immune profile, we also thoroughly examined the differences in the immune-related genes expression pattern between *TET1*-MUT and *TET1*-WT tumors (Fig. 6e). In line with immune infiltration and signatures, many stimulatory immunomodulators were generally upregulated in the *TET1*-MUT tumors, such as chemokines (*CXCL9*, *CXCL10*, *CCL5*) and cytolytic activity associated genes (*PRF1*, *GZMA*) (Additional file 7: Figure S5). Immune checkpoints, such as *CTLA4*, *LAG3*, and *TIGIT*, were also upregulated in *TET1*-MUT tumors against *TET1*-WT tumors.

These results indicated that *TET1*-MUT was strongly associated with enhanced tumor immunogenicity and relatively hot immune microenvironment, which firmly supported its predictive function to ICI treatment.

## Discussion

In this study based on carefully collected and curated genomic and clinical data, we observed that *TET1*-MUT was enriched in patients responding to ICIs and strongly predicted clinical benefit across multiple cancer types. *TET1*-MUT was found to be significantly associated with enhanced tumor immunogenicity and inflamed anti-tumor immunity. Notably, the predictive function of *TET1*-MUT was independent of TMB and MSI status, and also not attributed to its prognostic impact.

Although evidence concerning the association between DNA methylation and anti-immunity/immunotherapy is mounting [10–12], no clinical data regarding the correlation between genomic alterations of DNA methylation-related genes and response to ICIs are available. This study represents the first comprehensive report to examine the association between ICI response and specific mutated genes involving in the regulation of DNA methylation. Among 21 DNA methylation-related genes examined, *TET1* was found to be strongly associated with higher ORR, better DCB, longer PFS, and improved OS. These findings from real-world ICI-treated cohorts added great values to the robust link between DNA methylation and immunotherapy, and firmly supported the combination strategy of immunotherapy and epigenetic therapy [8].

Although the predictive value of *TET1*-MUT is remarkable, one may concern that its average occurrence frequency is relatively low (2.4%). However, its scope of application falls in a pan-cancer setting like MSI-H, thus there would still be substantial patients with *TET1*-MUT who are most likely to derive clinical benefit from ICI treatment. To date, MSI-H is the only pan-cancer biomarker approved by the FDA [4]. The pan-cancer occurrence frequency of MSI-H is about 4% [32]; but it is clustered in endometrial cancer, colorectal cancer, and gastric cancer while rarely detected in other cancers [33]. *TET1*-MUT is also more frequently detected in endometrial cancer and gastrointestinal cancer, as well as lung and skin cancers (Fig. 2b). On the other hand, the ORR in *TET1*-MUT patients is 60.9% (95% CI, 50.0 to 80.8%), which is numerically higher than that in MSI-H patients (about 32% ~ 55%) [34–37]; while no survival difference was observed between MSI-H and *TET1*-MUT patients (Fig. 4c). To sum up, the alteration frequency and predictive function of *TET1*-MUT are comparable to MSI-H. As *TET1*-MUT and MSI-H are not substantially overlapped (Fig. 4b), *TET1*-MUT is potential to serve as another pan-cancer biomarker to ICI response in addition to MSI-H.

TMB, PD-L1 expression, and T-cell inflamed GEP were all previously shown to be associated with clinical benefit in patients treated with ICIs [1, 3, 5, 6]. However, all of these three biomarkers are continuous variables without clearly defined cut points below which response does not occur and above which response is guaranteed [38]. And TMB and PD-L1 expression both vary largely among different detecting platforms and methods [39, 40]. In contrast, *TET1*-MUT is easily detected with next-generation sequencing assays, and its presence in the current analysis was strongly associated with ICI response. Therefore, prospective basket trial incorporating *TET1*-MUT as the biomarker is worth consideration. We plan to validate these findings prospectively in an upcoming randomized phase II study of a PD-1 antibody in multiple cancer types.

This retrospective analysis also has several limitations. Only five (*DNMT1*, *DNMT3A*, *DNMT3B*, *TET1*, *TET*2) out of the 21 DNA methylation-related genes are included in the MSK-IMPACT panel (Additional file 3: Table S1; *NTHL1* is only included in the 468-gene version). Consequently, the rest of genes could only be tested in part of the discovery cohort with WES data, of which the sample size is limited (*n* = 239). Thus we should not totally exclude the predictive function of these genes. Besides, although *TET1*-MUT was found to be strongly correlated with enhanced tumor immunogenicity and inflamed anti-tumor immunity, the underlying molecular mechanism of *TET1*-MUT sensitizing patients to ICI treatment still requires further exploration. Further elucidation of the molecular mechanism between *TET1*-MUT and ICI response would also help to make the combination strategy of epigenetic therapy and immunotherapy more precise.

## Conclusion

Our study provided solid evidence that *TET1*-MUT was associated with higher objective response rate, better durable clinical benefit, longer progression-free survival, and improved overall survival in patients receiving ICI treatment. Therefore, *TET1*-MUT can act as a novel predictive biomarker for immune checkpoint blockade across multiple cancer types. Further exploration of molecular mechanism and prospective clinical trials are warranted.

## Supplementary information

**Additional file 1: Figure S1.** Related to Fig. 2B_Flowchart of data processing for the pan-cancer alteration frequency analysis of *TET1*. (PDF 107 kb)

**Additional file 2: Figure S2.** Related to Fig. 6_Flowchart of data processing of the TCGA dataset. (PDF 107 kb)

**Additional file 3: Table S1.** Related to Fig. 2A_Key genes involving in the regulation of DNA methylation. (DOCX 16 kb)

**Additional file 4: Table S2.** Related to Fig. 3_ Patient characteristics between *TET1*-MUT and *TET1*-WT subgroups of the discovery cohort. (DOCX 16 kb)

**Additional file 5: Figure S3.** Related to Fig. 4_Kaplan-Meier curves investigating the prognostic impact of *TET1*-MUT in the TCGA cohort. (PDF 471 kb)

**Additional file 6: Figure S4.** Related to Fig. 6C_The differences of tumor-infiltrating leukocytes between *TET1*-MUT and *TET1*-WT tumors. (Mann-Whitney U test with Bonferroni correction. *, *P* < 0.05; **, *P* < 0.01; ***, *P* < 0.001). (PDF 893 kb)

**Additional file 7: Figure S5.** Related to Fig. 6E_ The expression levels of immune-related genes, such as chemokines (A), cytolytic activity associated genes (B) and immune checkpoints (C) in *TET1*-MUT tumors versus *TET1*-WT tumors. (Mann-Whitney U test with Bonferroni correction. *, *P* < 0.05; **, *P* < 0.01; ***, *P* < 0.001). (PDF 527 kb)

## Data Availability

All of the data we used in this study were publicly available as described in the Method section.

## Abbreviations

BLCA: Bladder urothelial carcinoma
BRCA: Breast cancer
CESC: Cervical squamous-cell carcinoma and endocervical adenocarcinoma
CI: Confidence interval
CR: Complete response
CRC: Colorectal cancer
CTLA-4: Cytotoxic T lymphocyte antigen 4
DCB: Durable clinical benefit
FDA: Food and Drug Administration
FDR: False discovery rate
FPKM: Fragments Per Kilobase of exon model per Million mapped fragments
GEP: Gene expression profile
HNSCC: Head and neck squamous cell carcinoma
HR: Hazard ratio
ICIs: Immune checkpoint inhibitors
IRB: Institutional Review Board
KIRP: Kidney renal papillary cell carcinoma
LIHC: Liver hepatocellular carcinoma
Mb: Megabase
MSI-H: high microsatellite instability
MSI-L: low microsatellite instability
MSS: microsatellite stable
NDB: No durable benefit
NE: Not evaluable
NSCLC: Non-small-cell lung cancer
ORR: Objective response rate
OS: Overall survival
PD: Progression of disease
PD-(L)1: Programmed cell death (ligand) 1
PFS: Progression-free survival
PR: Partial response
RECIST: Response Evaluation Criteria in Solid Tumors
SD: Stable disease
SKCM: Skin cutaneous melanoma
ssGSEA: Single sample gene set enrichment
STAD: Stomach adenocarcinoma
TCGA: The Cancer Genome Atlas
*TET1*-MUT: *TET1*-mutant
*TET1*-WT: *TET1*-wildtype
TMB: Tumor mutational burden
UCEC: Uterine corpus endometrial carcinoma
WES: Whole-exome sequencing
