# Supplementary material for: Alteration in TET1 as potential biomarker for immune checkpoint blockade in multiple cancers
Source: J Immunother Cancer. 2019 Oct 17;7:264. doi: 10.1186/s40425-019-0737-3 (PMC6798429; doi:10.1186/s40425-019-0737-3)
Supplement: Supplementary file 1 — Additional file 1: Figure S1. Related to Fig. 2B_Flowchart of data processing for the pan-cancer alteration frequency analysis of TET1. (PDF 107 kb) [file 40425_2019_737_MOESM1_ESM.pdf]

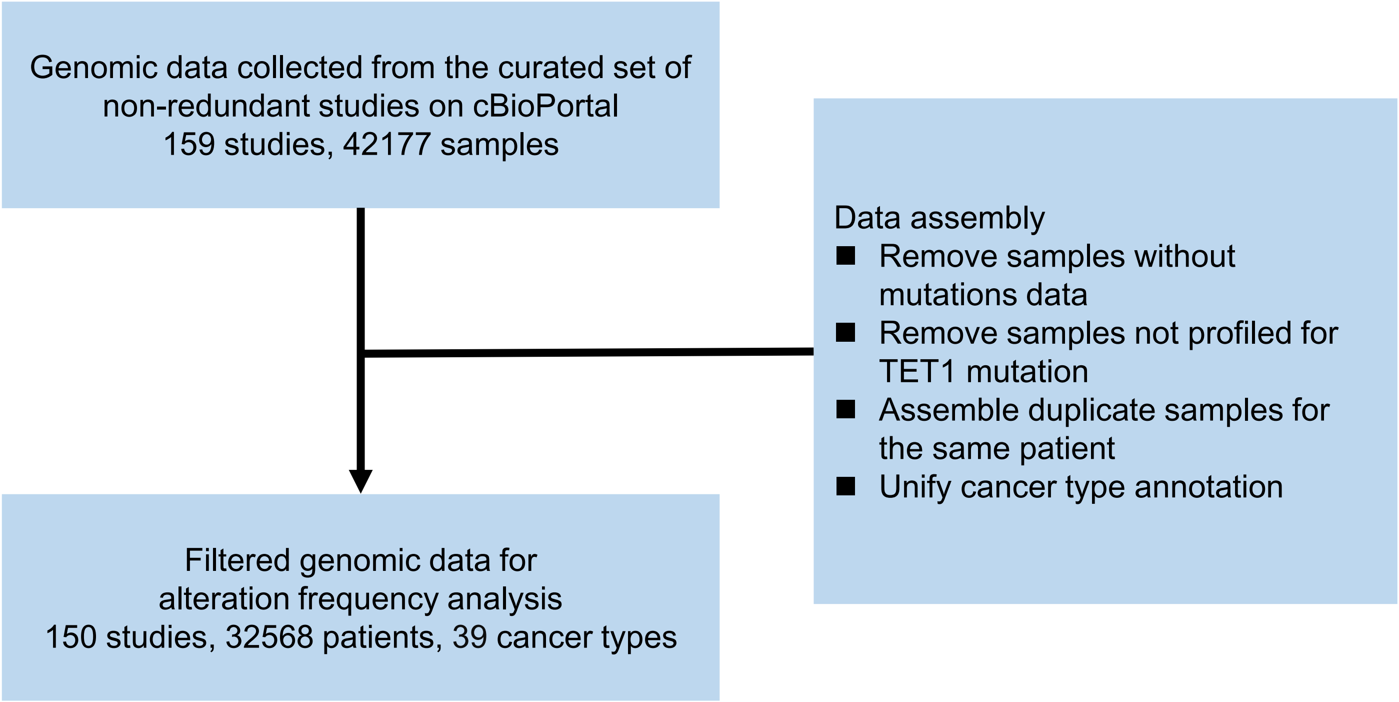
Genomic data collected from the curated set of non-redundant studies on cBioPortal
159 studies, 42177 samples
Data assembly
Remove samples without mutations data
Remove samples not profiled for TET1 mutation
Assemble duplicate samples for the same patient
Unify cancer type annotation
Filtered genomic data for alteration frequency analysis
150 studies, 32568 patients, 39 cancer types
