# Supplementary material for: Alteration in TET1 as potential biomarker for immune checkpoint blockade in multiple cancers
Source: J Immunother Cancer. 2019 Oct 17;7:264. doi: 10.1186/s40425-019-0737-3 (PMC6798429; doi:10.1186/s40425-019-0737-3)
Supplement: Supplementary file 2 — Additional file 2: Figure S2. Related to Fig. 6_Flowchart of data processing of the TCGA dataset. (PDF 107 kb) [file 40425_2019_737_MOESM2_ESM.pdf]

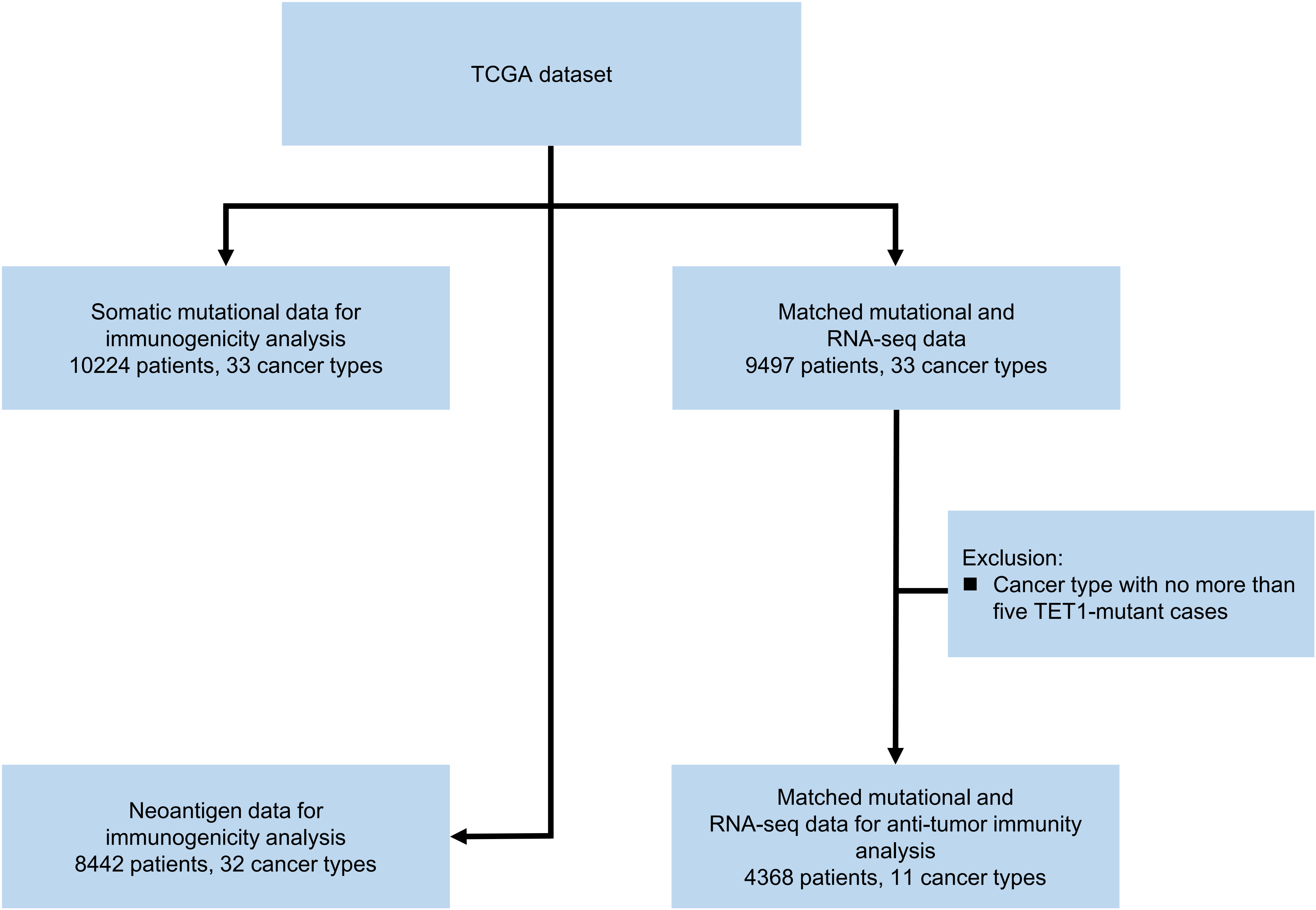
TCGA dataset
Somatic mutational data for immunogenicity analysis
10224 patients, 33 cancer types
Matched mutational and RNA-seq data
9497 patients, 33 cancer types
Exclusion:
Cancer type with no more than five TET1-mutant cases
Neoantigen data for immunogenicity analysis
8442 patients, 32 cancer types
Matched mutational and RNA-seq data for anti-tumor immunity analysis
4368 patients, 11 cancer types
