# Supplementary material for: Alteration in TET1 as potential biomarker for immune checkpoint blockade in multiple cancers
Source: J Immunother Cancer. 2019 Oct 17;7:264. doi: 10.1186/s40425-019-0737-3 (PMC6798429; doi:10.1186/s40425-019-0737-3)
Supplement: Supplementary file 3 — Additional file 3: Table S1. Related to Fig. 2A_Key genes involving in the regulation of DNA methylation. (DOCX 16 kb) [file 40425_2019_737_MOESM3_ESM.docx]

**Table S1. Key genes involving in the regulation of DNA methylation.**

| **Gene Symbol** | **Gene Name** | **Function** | **MSK-IMPACT panel^#^** | **Cases with gene mutations** | **Total cases** | **Proportion (%)** | ***P* value^*^**  **(FDR adjusted)** |
| --- | --- | --- | --- | --- | --- | --- | --- |
| *DNMT1* | DNA methyltransferase 1 | Writer | Included | 13 | 519 | 2.50% | 1.000 |
| *DNMT3A* | DNA methyltransferase 3A | Writer | Included | 13 | 519 | 2.50% | 1.000 |
| *DNMT3B* | DNA methyltransferase 3B | Writer | Included | 13 | 519 | 2.50% | 1.000 |
| *TET1* | Tet methylcytosine dioxygenase 1 | Eraser | Included | 23 | 519 | 4.43% | **0.003** |
| *TET2* | Tet methylcytosine dioxygenase 2 | Eraser | Included | 30 | 519 | 5.78% | 0.753 |
| *TET3* | Tet methylcytosine dioxygenase 3 | Eraser | Not included | 18 | 239 | 7.53% | 1.000 |
| *TDG* | Thymine DNA glycosylase | Mediator | Not included | 6 | 239 | 2.51% | 1.000 |
| *SMUG1* | Single-strand-specific monofunctional uracil-DNA glycosylase 1 | Mediator | Not included | 1 | 239 | 0.42% | 1.000 |
| *UNG* | Uracil DNA glycosylase | Mediator | Not included | 1 | 239 | 0.42% | 1.000 |
| *NEIL1* | Endonuclease VIII-like glycosylase 1 | Mediator | Not included | 1 | 239 | 0.42% | 1.000 |
| *NTHL1* | Endonuclease III-like 1 | Mediator | Included | 2 | 261 | 0.77% | 1.000 |
| *MeCP2* | Methyl CpG binding protein 2 | Mediator | Not included | 0 | 239 | 0.00% | NA |
| *MBD1* | Methyl-CpG binding domain protein 1 | Mediator | Not included | 5 | 239 | 2.09% | 1.000 |
| *MBD2* | Methyl-CpG binding domain protein 2 | Mediator | Not included | 0 | 239 | 0.00% | NA |
| *MBD3* | Methyl-CpG binding domain protein 3 | Mediator | Not included | 4 | 239 | 1.67% | 0.641 |
| *MBD4* | Methyl-CpG binding domain protein 4 | Mediator | Not included | 6 | 239 | 2.51% | 1.000 |
| *ZBTB33* | zinc finger and BTB domain containing 33 | Mediator | Not included | 3 | 239 | 1.26% | 1.000 |
| *ZBTB38* | zinc finger and BTB domain containing 38 | Mediator | Not included | 6 | 239 | 2.51% | 1.000 |
| *ZBTB4* | zinc finger and BTB domain containing 4 | Mediator | Not included | 9 | 239 | 3.77% | 1.000 |
| *UHRF1* | Ubiquitin-like with PHD and ring finger domains 1 | Mediator | Not included | 0 | 239 | 0.00% | NA |
| *UHRF2* | Ubiquitin-like with PHD and ring finger domains 2 | Mediator | Not included | 5 | 239 | 2.09% | 1.000 |

**#** Indicated that whether the genes were included in the MSK-IMPACT panel. Notably, *NTHL1* was only included in the 468-gene version of MSK-IMPACT panel.

***** *P* value < 0.05 denoted significantly enrichment in patients responding to immune checkpoint inhibitos.

FDR, False discovery rate.
