# Supplementary material for: Alteration in TET1 as potential biomarker for immune checkpoint blockade in multiple cancers
Source: J Immunother Cancer. 2019 Oct 17;7:264. doi: 10.1186/s40425-019-0737-3 (PMC6798429; doi:10.1186/s40425-019-0737-3)
Supplement: Supplementary file 4 — Additional file 4: Table S2. Related to Fig. 3_ Patient characteristics between TET1-MUT and TET1-WT subgroups of the discovery cohort. (DOCX 16 kb) [file 40425_2019_737_MOESM4_ESM.docx]

**Table S2. Patient characteristics between *TET1*-MUT and *TET1*-WT subgroups of the discovery cohort.**

| **Characteristics** | ***TET1* status (No. [%]^#^)** | | ***P* value^*^** |
| --- | --- | --- | --- |
|  | **Wildtype** | **Mutant** |  |
| **No. of patients** | 496 (95.6) | 23 (4.4) | - |
| **Median age, years (IQR)** | 64 (55, 71) | 65 (57, 72) | 0.663 |
| **Sex** |  |  | 0.523 |
| Male | 285 (95.0) | 15 (5.0) |  |
| Female | 211 (96.3) | 8 (3.7) |  |
| **Cancer type** |  |  | 0.500 |
| Bladder cancer | 25 (92.6) | 2 (7.4) |  |
| Esophagogastric cancer | 38 (95.0) | 2 (5.0) |  |
| Head and neck cancer | 10 (100.0) | 0 (0.0) |  |
| Melanoma | 139 (93.9) | 9 (6.1) |  |
| Non-small-cell lung cancer | 284 (96.6) | 10 (3.4) |  |
| **Drug class** |  |  | 0.105 |
| CTLA-4, monotherapy | 134 (94.4) | 8 (5.6) |  |
| PD-(L)1, monotherapy | 310 (96.9) | 10 (3.1) |  |
| CTLA-4 + PD-(L)1, combination therapy | 52 (91.2) | 5 (8.8) |  |
| **Best overall response** |  |  | 0.002 |
| CR/PR | 112 (88.9) | 14 (11.1) |  |
| SD | 134 (97.8) | 3 (2.2) |  |
| PD | 246 (97.6) | 6 (2.4) |  |
| NE | 4 (100.0) | 0 (0.0) |  |
| **Durable clinical benefit** |  |  | 0.001 |
| DCB | 150 (90.9) | 15 (9.1) |  |
| NDB | 324 (98.2) | 6 (1.8) |  |
| NE | 22 (91.7) | 2 (8.3) |  |
| **Median TMB, Mutation/Mb (IQR)** | 6.72 (3.73, 12.26) | 23.20 (11.92, 41.46) | <0.001 |

^#^ Indicated percentage of patients with *TET1*-MUT amongst total patients within a given category (i.e. a specific cancer type, or a specific drug class).

* Student’s t test for continous variables and fisher’s exact test for categorical variables. *P* value < 0.05 denoted statistically significant differences in a given patient characteristics between *TET1*-MUT and *TET1*-WT subgroups.

Abbreviations: CR, complete response; CTLA-4, cytotoxic T-cell lymphocyte-4; DCB, durable clinical benefit; IQR, interquartile range; Mb, megabase; NDB, no durable benefit; NE, not evaluable; PD, progressive disease; PD-(L)1, programmed cell death-1 or programmed death-ligand 1; PR, partial response; SD, stable disease; TMB, tumor mutational burden.
