# Supplementary figures and images for: Alteration in TET1 as potential biomarker for immune checkpoint blockade in multiple cancers
Source: J Immunother Cancer. 2019 Oct 17;7:264. doi: 10.1186/s40425-019-0737-3 (PMC6798429; doi:10.1186/s40425-019-0737-3)

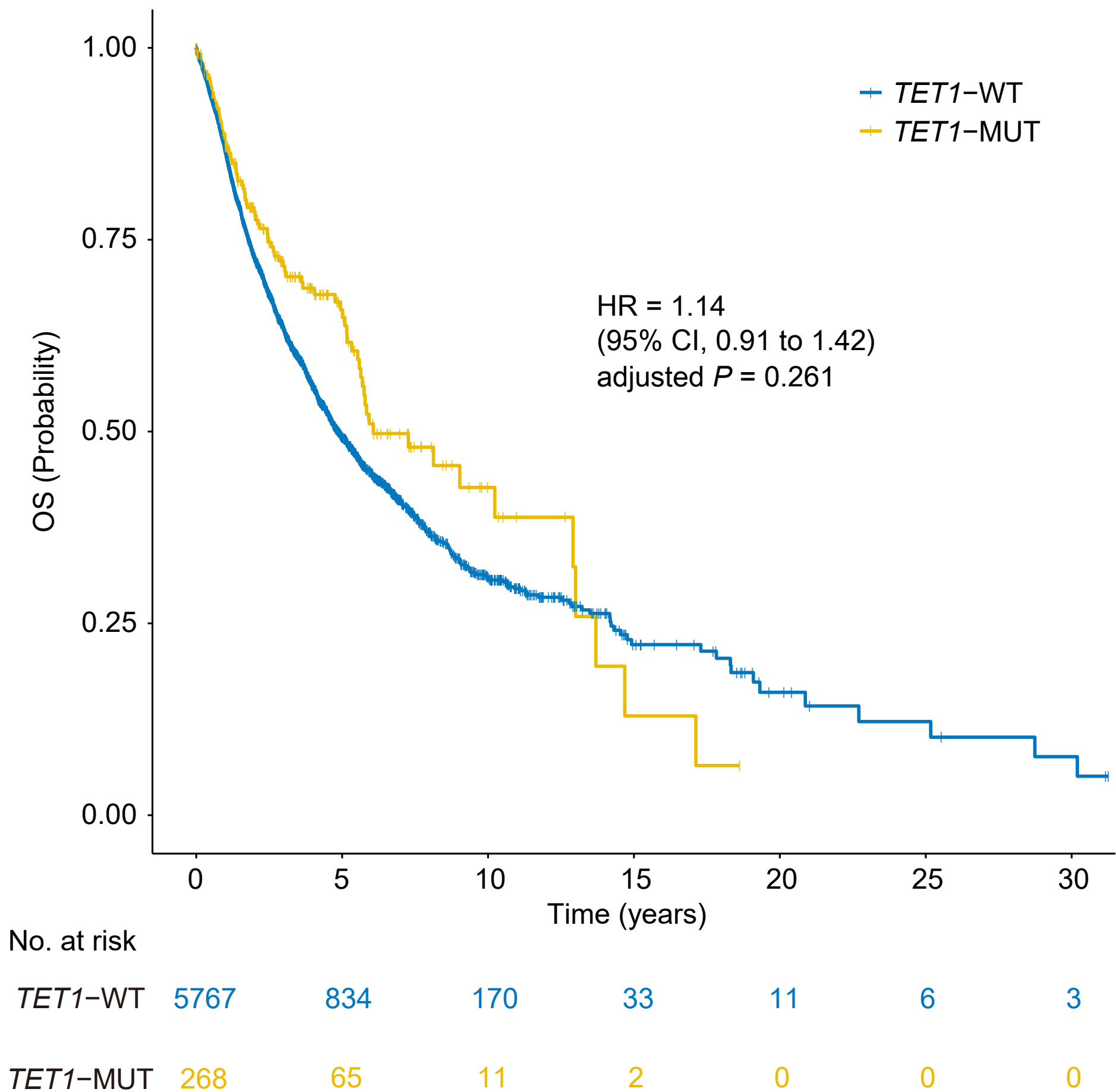

Supplement: Supplementary file 5 — Additional file 5: Figure S3. Related to Fig. 4_Kaplan-Meier curves investigating the prognostic impact of TET1-MUT in the TCGA cohort. (PDF 471 kb) [file 40425_2019_737_MOESM5_ESM.pdf]

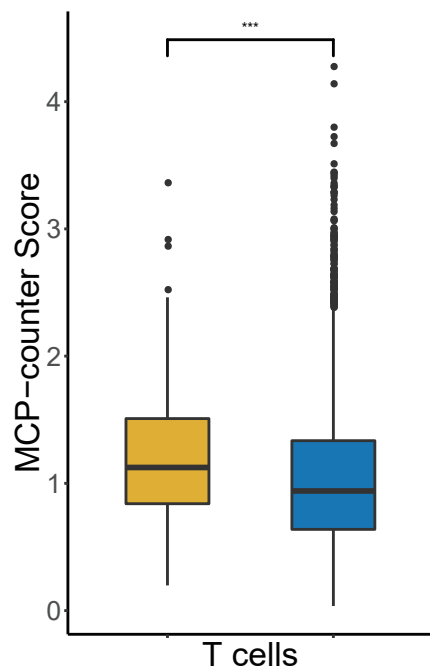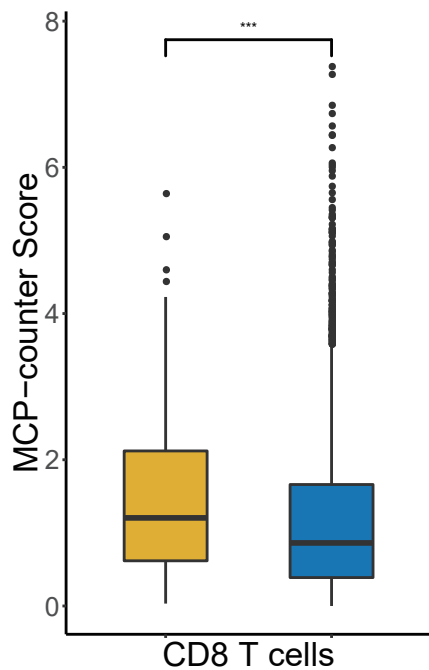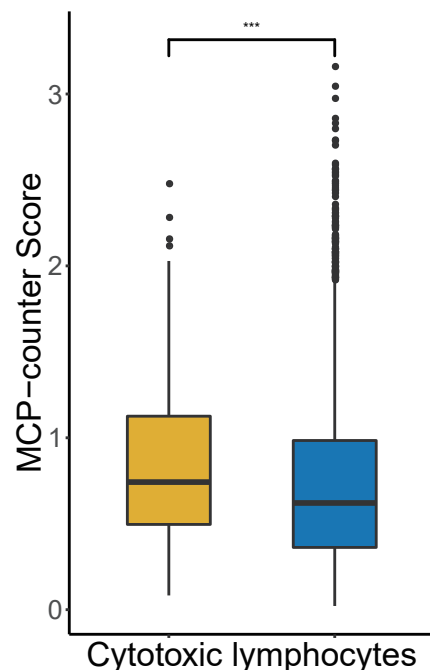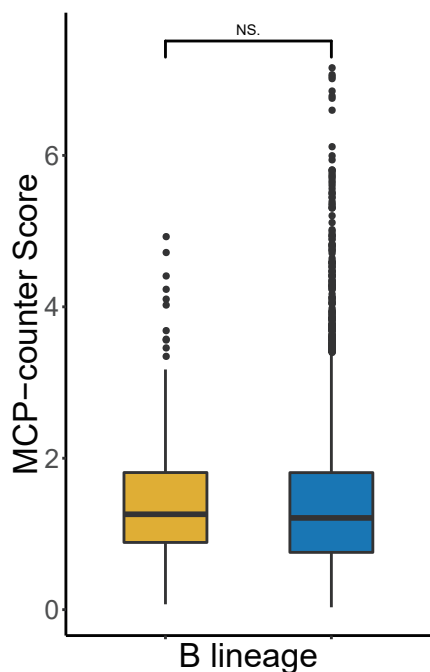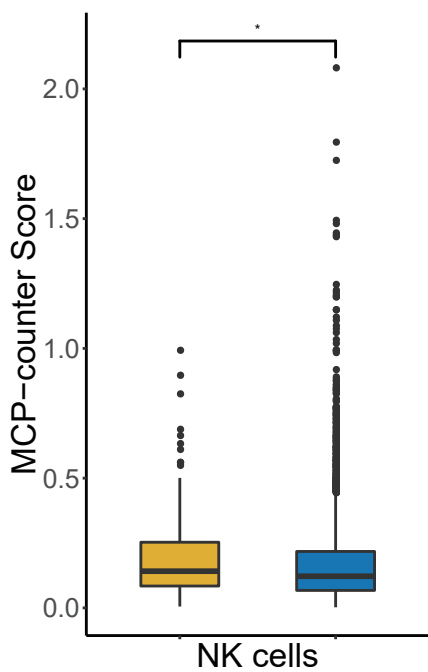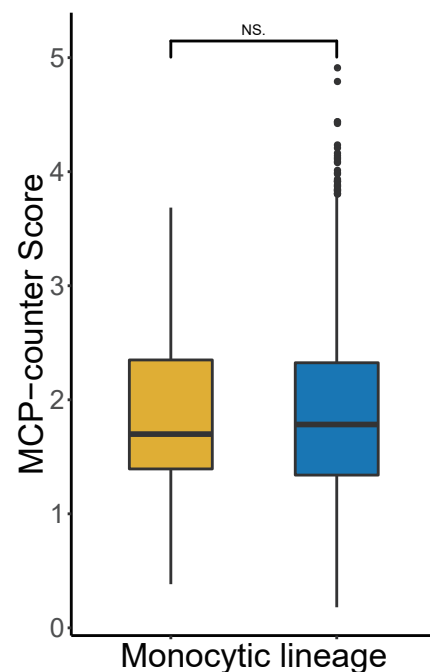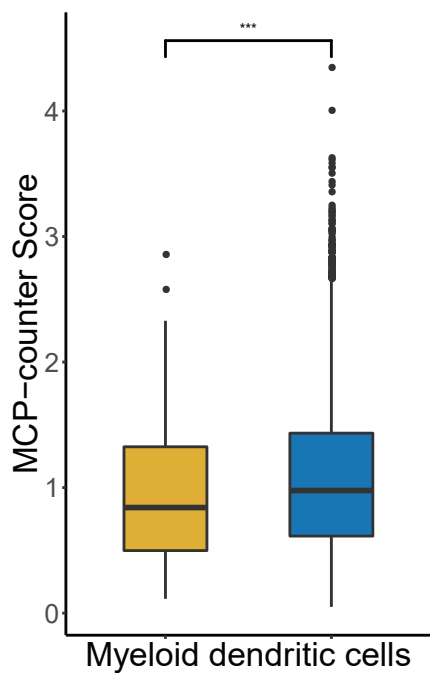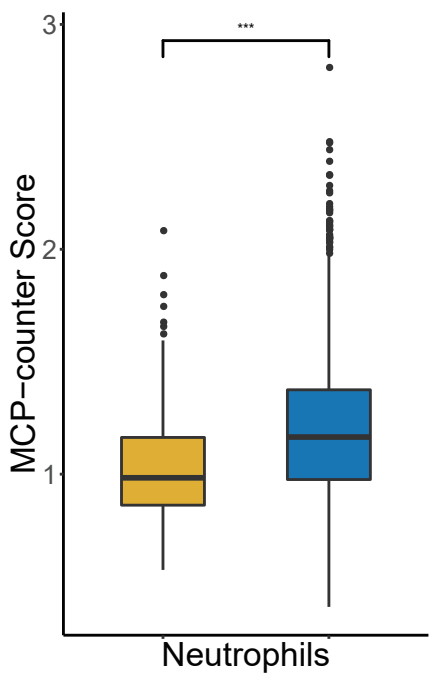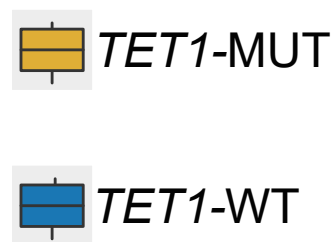

Supplement: Supplementary file 6 — Additional file 6: Figure S4. Related to Fig. 6C_The differences of tumor-infiltrating leukocytes between TET1-MUT and TET1-WT tumors. (Mann-Whitney U test with Bonferroni correction. *, P < 0.05; **, P < 0.01; ***, P < 0.001). (PDF 893 kb) [file 40425_2019_737_MOESM6_ESM.pdf]

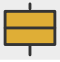 *TET1-MUT*      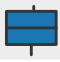 *TET1-WT*

A

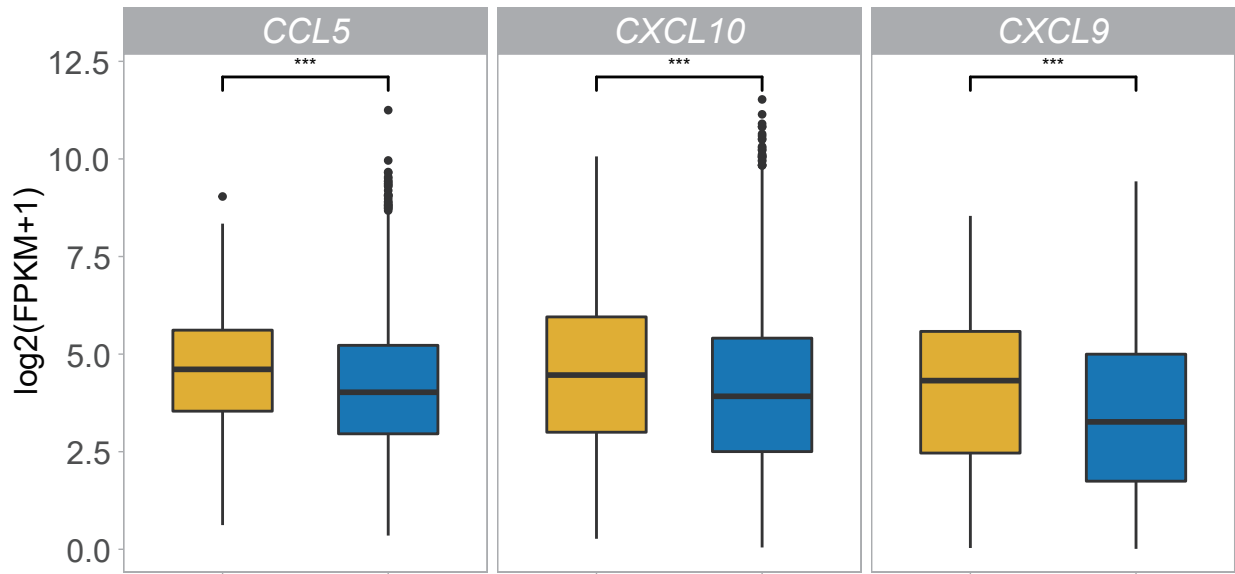

B

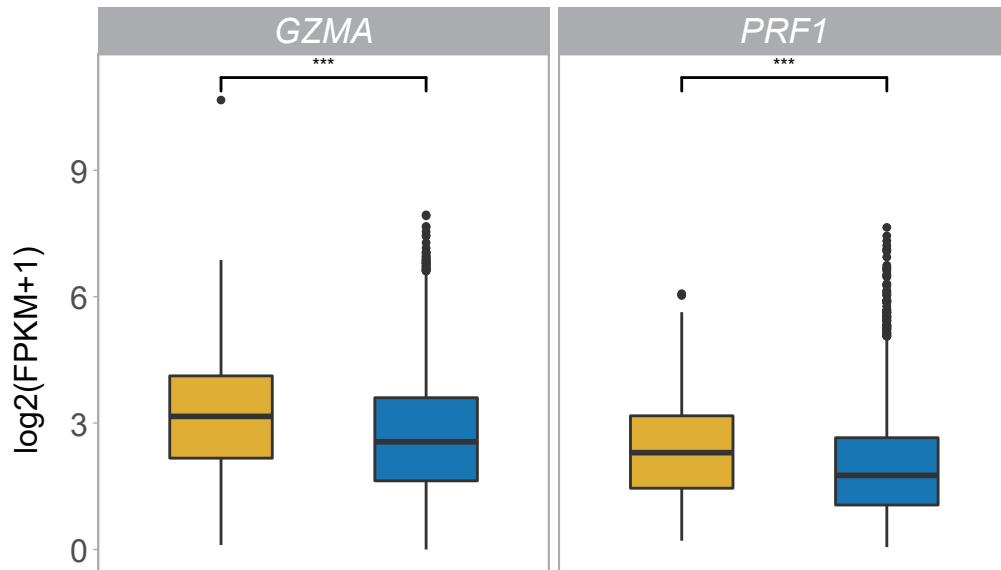

C

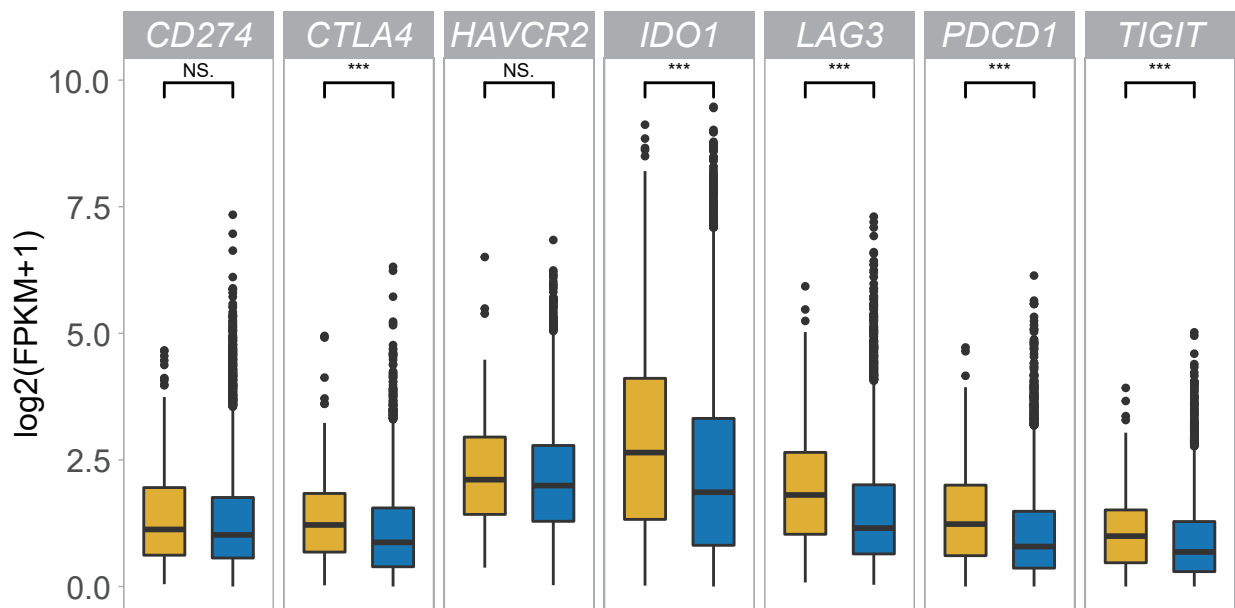

Supplement: Supplementary file 7 — Additional file 7: Figure S5. Related to Fig. 6E_ The expression levels of immune-related genes, such as chemokines (A), cytolytic activity associated genes (B) and immune checkpoints (C) in TET1-MUT tumors versus TET1-WT tumors. (Mann-Whitney U test with Bonferroni correction. *, P < 0.05; **, P < 0.01; ***, P < 0.001). (PDF 527 kb) [file 40425_2019_737_MOESM7_ESM.pdf]
